# Supplementary material for: Insulin as Monotherapy and in Combination with Other Glucose-Lowering Drugs Is Related to Increased Risk of Diagnosis of Pneumonia: A Longitudinal Assessment over Two Years
Source: J Pers Med. 2021 Sep 29;11(10):984. doi: 10.3390/jpm11100984 (PMC8537451; doi:10.3390/jpm11100984)
Supplement: Supplementary file 1 [file jpm-11-00984-s001.zip › jpm-1359048-supplementary.pdf]

Supplemental material

Baseline comorbidity table

We observe patients' comorbidities in the baseline period in all therapy groups and the control group. Comorbidities are defined using ICD-10 codes as follows: essential hypertension (aHTN, I10); ischemic heart diseases (CVD, I20–I25); cerebral infarction (Stroke, I63-I64); diseases of arteries, arterioles and capillaries (pAVK, I70–I79); acute kidney failure and chronic kidney disease (KD, N17–N19), overweight and obesity (OBES, E66); nicotine dependence (F17); polyneuropathies and other disorders of the peripheral nervous system (PNP, G60–G64); retinal disorders in diseases classified elsewhere (H36); depression (F32, F33); disorders of lipoprotein metabolism and other lipidemias (LIP, E78).

Table S1. Patient baseline comorbidities for therapy groups and control group.

**Therapy group**	**aHTN [%]**	**CVD [%]**	**Stroke [%]**	**pAVK [%]**	**KD [%]**	**OBES [%]**	**LIP [%]**	**PNP [%]**
MET	19.77	3	0.62	1.82	1.25	4.46	2.74	0.88
MET+Statins	30.14	13.4	1.89	4.98	1.86	6.21	15.38	1.07
Statins	33.73	18.13	2.2	6.59	5.94	4.78	19.73	1.32
Insulin	21.37	5.56	1.05	5.46	5.27	2.98	3.79	2.63
Insulin+Statins	38.52	19.5	2.52	11.98	12.04	7.38	19.26	3.1
MET+Insulin+Statins	34.77	17.29	2.32	10.03	2.94	9.9	19.74	3.2
MET+SU	18.27	3.12	0.36	1.87	1.07	2.85	2.76	0.98
SU	22.64	3.76	1.47	3.67	4.67	3.94	3.12	1.01
MET+SU+Statins	24.15	10.43	2.27	5.9	2.04	2.95	11.68	0.68
MET+Insulin	25.48	5.54	0.69	4.16	2.35	7.2	4.43	2.08
SU+Statins	30.45	13.51	1.98	5.95	6.49	3.78	15.32	1.8
MET+TZD+Statins	20.9	6.97	2.99	5.97	1.99	4.48	12.44	1.99
DPP-IV	28.8	3.66	0.52	2.62	3.66	6.28	5.24	1.57
MET+DPP-IV+Statins	33.86	11.11	1.06	7.41	1.06	8.99	14.81	1.06
DPP-IV+Statins	36.07	18.58	1.64	5.46	13.66	9.84	16.39	3.28
MET+TZD	13.29	1.27	0.63	3.8	2.53	4.43	2.53	0.63
DPP-IV+SU	22.86	5.71	0	3.57	8.57	3.57	4.29	2.14
DPP-IV+SU+Statins	33.58	14.93	3.73	8.21	11.19	5.22	17.91	0.75
MET+DPP-IV	16.79	6.57	1.46	2.92	0	5.11	2.19	1.46
TZD	22.41	1.72	0.86	0	6.03	6.9	1.72	0.86
TZD+Statins	31.43	15.24	2.86	5.71	6.67	8.57	20.95	1.9
MET+Other-AD	18.75	2.08	1.04	4.17	2.08	4.17	2.08	0
DPP-IV+Insulin+Statins	50	21.74	4.35	18.48	22.83	21.74	29.35	5.43
TZD-SU	32.56	5.81	1.16	4.65	5.81	8.14	6.98	2.33
TZD+SU+Statins	24.36	11.54	0	3.85	7.69	8.97	15.38	1.28
MET+TZD+SU	20	2.86	1.43	4.29	5.71	7.14	2.86	0
Other-AD+Statins	30.65	16.13	4.84	16.13	9.68	1.61	4.84	0
Alpha-Gluc.-Inh.	17.39	6.52	0	6.52	4.35	4.35	2.17	0
Alpha-Gluc.-Inh.+Statins	29.73	8.11	2.7	8.11	8.11	5.41	13.51	2.7

DPP-IV+Insulin	48.39	25.81	0	6.45	25.81	29.03	19.35	16.13
Insulin+SU	23.08	0	0	3.85	7.69	3.85	7.69	3.85
MET+Alpha-Gluc.-Inh.	8.7	0	0	0	0	0	4.35	0
DPP-IV+ Other-AD+Statins	38.46	15.38	0	0	0	7.69	15.38	0
MET+ Alpha-Gluc.-Inh.+Statins	27.27	18.18	9.09	0	0	0	36.36	0
DPP-IV+Other-AD	40	0	0	0	10	0	0	0
Controls	26.14	5.79	0.94	2.83	4.75	4.38	4.54	0.79

Supplementary Table S1: Patient baseline comorbidities for therapy groups and control group. MET=metformin, SU=sulfonylureas, TZD= Thiazolidinediones, DPP-IV=DPP-IV inhibitors, other-AD=other blood-glucose-lowering drugs, Alpha-Gluc.-Inh.= alpha-glucosidase inhibitors).
